# Current radiotracers to image neurodegenerative diseases

**DOI:** 10.1186/s41181-019-0070-7

**Published:** 2019-07-26

**Authors:** Solveig Tiepolt, Marianne Patt, Gayane Aghakhanyan, Philipp M. Meyer, Swen Hesse, Henryk Barthel, Osama Sabri

**Affiliations:** Department of Nuclear Medicine, University of Leipzig, Liebigstraße 18, 04103 Leipzig, Germany

**Keywords:** Alzheimer’s disease, Parkinsonian syndromes, Primary progressive aphasia, Frontotemporal dementia, PET, SPECT, ß-amyloid, Tau, Cholinergic system, Dopaminergic system

## Abstract

The term of neurodegenerative diseases covers a heterogeneous group of disorders that are distinguished by progressive degeneration of the structure and function of the nervous system such as dementias, movement disorders, motor neuron disorders, as well as some prion disorders. In recent years, a paradigm shift started for the diagnosis of neurodegenerative diseases, for which successively clinical testing is supplemented by biomarker information. In research scenarios, it was even proposed recently to substitute the current syndromic by a biological definition of Alzheimer’s diseases. PET examinations with various radiotracers play an important role in providing non-invasive biomarkers and co-morbidity information in neurodegeneration. Information on co-morbidity, e.g. Aβ plaques and Lewy-bodies or Aβ plaques in patients with aphasia or the absence of Aβ plaques in clinical AD patients are of interest to expand our knowledge about the pathogenesis of different phenotypically defined neurodegenerative diseases. Moreover, this information is also important in therapeutic trials targeting histopathological abnormalities.

The aim of this review is to present an overview of the currently available radiotracers for imaging neurodegenerative diseases in research and in routine clinical settings. In this context, we also provide a short summary of the most frequent neurodegenerative diseases from a nuclear medicine point of view, their clinical and pathophysiological as well as nuclear imaging characteristics, and the resulting need for new radiotracers.

## Introduction

It is well recognized that increased life expectancy results in an increased frequency of neurodegenerative diseases. In the last two to three decades, the development of new diagnostic and therapeutic methods has been intensified in neurodegenerative diseases. Molecular radiopharmaceutical-based neuroimaging is a growing field and provides several new diagnostic methods to investigate and characterize neurodegenerative diseases during life. This review summarizes the facts for the most frequent neurodegenerative diseases from a nuclear medicine point of view. Furthermore, the review provides information on approved radiotracers and ongoing research activities in the development of new radiotracers for imaging neurodegenerative diseases as well as a short passage about the need for novel radiotracers.

## Neurodegenerative diseases – important histopathological and clinical facts

### Alzheimer’s disease (AD)

Alzheimer’s disease (AD) is the most common neurodegenerative disease causing dementia in the elderly. Histopathological characteristics of this disease are a progressive accumulation of β-amyloid (Aβ) plaques and hyperphosphorylated neurofibrillary tau protein (tau). However, only approximately 50 % of the patients have solely Alzheimer’s pathology. Many patients show additional pathologic changes related to other neurodegenerative diseases in autopsy studies (Alzheimer’s Association report 2018 - https://www.sciencedirect.com/science/article/pii/S1552526018300414). Along with the typical clinical features like memory impairment, especially in the semantic and episodic domain and the executive dysfunction (McKhann et al. 2011), atypical variants of AD exist. As such, posterior cortical atrophy (PCA) and logopenic variant primary progressive aphasia (lvPPA) are labeled as atypical AD, since the histopathological changes (i.e. Aβ and tau accumulation) in these neurodegenerative diseases determine the “typical” AD features, although the distributional pattern of the pathologic changes seems to be different (Crutch et al. 2012; Harris and Jones 2014). However, in both PCA and lvPPA a significant minority of cases showed other underlying pathologies than AD, e.g. Lewy bodies, transactive response DNA binding protein of about 43 kDa (TAR DNA-binding protein 43, TDP-43) proteinopathies, “pure” tauopathy or cerebrovascular disease (Crutch et al. 2012; Harris and Jones 2014) (Table 1). The core clinical features of patients with PCA are caused by a decline in visual processing and other posterior cognitive functions, e.g. space and/or object perception deficits, simultanagnosia or constructional dyspraxia (Crutch et al. 2017). The core clinical features of lvPPA are impaired single-word retrieval in spontaneous speech and naming as well as impaired repetition of sentences and phrases (Harris and Jones 2014).

**Table 1:** Summary of histopathological findings of the different neurodegenerative syndromes

Disease	Aβ plaques	Tau deposits	α- synuclein	TDP-43	Other pathologies
AD	++ (in up to 90%)^l^	++ 3R/4R	–	–	–
PCA	++ (in up to 78%)^a,c^	++ 3R/4R (AD in ≈ 76%), 4R (CBD in ≈ 9,5%)^c^	+ Lewy-bodies (in ≈ 14%)^c^	–	(+) Prion-associated diseases
lvPPA	++ (in up to 56%)^b^	++ 3R/4R (AD in up to 56%)^b^, other subtypes (in ≈ 10%)^b^	(+) Lewy-bodies (< 10%)^e^	+ (in up to 25%, mainly type A)^d^,	(+) CJD
bvFTD	+ (in up to 13%)^f^	+ 4R (CBD in up to ≈ 9%)^h^ 4R (PSP ≈ 8%)^h^ 3R (PiD in ≈ 7%)^h^ 3R/4R (AD in ≈ 13%)^h^	–	++ (type A in ≈8%, type B in ≈23%, type C in ≈7% type U in ≈ 10%)^h^	(+) FTLD-FUS in ≈7%^h^
svPPA	+ (in up to 14%)^b^	+ 3R (PiD in up to 15%)^b^	–	++ (in up to 86%, pre-dominantly type C)^b^	–
nfvPPA	+ (in up to 12%)^g^	++ 4R (CBD in up to ≈ 54%)^h^ 4R (PSP ≈ 18%)^g,h^ 3R (PiD in ≈ 12%)^g^ 3R/4R (AD in ≈ 12%)^g^	(+) Lewy-bodies (< 10%)^b,g^	+ (type A in up to 18%) ^g,h^	–
PD/DLB	++ (PD in up to 15%, DLB in up to 80%)^k^	(+) 4R (PSP in ≈ 8%)^i^ 4R (CBD in ≈ 2%)^i^	++ Lewy bodies (in up to 77%)^i,j^ α- synuclein (MSA in ≈5%)^i^	–	–
HD	–	+ 3R/4R	–	++ Huntingtin	–

*Aβ* β-amyloid, *AD* Alzheimer’s disease, *bvFTD* Behavioural variant frontotemporal dementia, *CBD* Corticobasal degeneration, *CJD* Creutzfeld Jacob disease, *FTLD-FUS* Frontotemporal lobar degeneration-fused in sarcoma, *lvPPA* Logopenic variant primary progressive aphasia, *MSA* Multisystem atrophy, *PCC* Posterior cingulate cortex, *PiD* Pick’s disease, *PSP* Progressive supranuclear palsy, *svPPA* Semantic variant primary progressive aphasia, *TDP* Transactive response DNA binding protein of about 43 kDa, *3R* three repeat tau isoform, *4R* four repeat tau isoform

**++** Frequently occurring; **+** Sometimes occurring; **(+)** Rarely occurring; **−** Not occurring

^a^(Tang-Wai et al. 2004) ^b^(Harris and Jones 2014) ^c^(Renner et al. 2004) ^d^(Rogalski et al. 2014) ^e^(Harris et al. 2013) ^f^ (Perry et al. 2017) ^g^ (Mesulam et al. 2014) ^h^ (Caso et al. 2014) ^i^ (Dickson 2018) ^j^ (Skogseth et al. 2017) ^k^ (Drzezga 2010) ^l^(Jack et al. 2018)

### Frontotemporal lobar degeneration (FTLD)

FTLD is a potpourri of clinically, histopathologically and genetically different disorders that become united due to predominant pathological involvement of the frontal and temporal brain regions. Three distinct clinical phenotypes of FTLD are recognized including a behavior/dysexecutive syndrome - the behavioral variant of frontotemporal dementia (bvFTD); language disorders - the primary progressive aphasia (PPA): semantic variant (svPPA) and non-fluent/agrammatic variant (nfvPPA); and motor disorders (amyotrophic lateral sclerosis, corticobasal and progressive supranuclear palsy syndromes).

In general, an individual FTLD disorder can be ascribed to different histopathologies such as tauopathy (FTLD-tau), TDP-43 proteinopathy (FTLD-TDP-43) and the FET protein family that consists of Fused in sarcoma, Ewing sarcoma and TATA-binding protein associated factor 15 proteinopathy (FTLD-FET) (Table 1). Here, some histopathology is more often seen in one than in another FTLD disorder (Harris and Jones 2014; Bang et al. 2015; Mackenzie and Neumann 2016). Thus, more than 70% of the patients with svPPA have TDP-43 proteinopathy. Over 50% of patients with nfvPPA have FTLD-tau, approximately 20% show TDP-43 proteinopathy (Harris and Jones 2014). Almost half of the patients with bvFTD have FTLD-tau (Pressman and Miller 2014) and more than 25% TDP-43 pathology (Bang et al. 2015). Of interest, tauopathies can be differentiated in at least 5 subtypes according to their molecular subtype (Mackenzie and Neumann 2016).

### Parkinsonian syndromes

In Parkinson’s disease (PD), a degeneration of the nigrostriatal system occurs causing a reduction of the neurotransmitter dopamine. Degeneration of dopaminergic neurons in the substantia nigra pars compacta is an inherent neuropathological sign of PD. Histologically, most patients suffering from PD exhibit intracellular accumulation of protein inclusions mainly constituted of α-synuclein (Lewy bodies). However, some patients with specific genetic forms of PD do not have Lewy body pathology and it remains unclear how Lewy bodies and neuronal loss are connected to each other. The typical clinical criteria of PD consist of symptoms such as bradykinesia, rigidity or resting tremor as cardinal motor manifestations (Postuma et al. 2015). Important supportive criteria include response to dopamine replacement, unilateral onset, olfactory dysfunction and REM sleep behavior disorder (Berg et al. 2013; Postuma et al. 2015). Corticobasal degeneration (CBD), progressive supranuclear palsy (PSP) and multisystem atrophy (MSA) are classified as atypical parkinsonisms. Histopathologically, CBD and PSP belong to primary tauopathies while MSA, DLB and PD are characterized by a pathologic accumulation of α-synuclein protein (α-synucleinopathies). The clinical presentation of CBD can be subdivided into 4 phenotypes: corticobasal syndrome, behavior spatial syndrome, nfvPPA and PSP (Armstrong et al. 2013) demonstrating that the accuracy to diagnose CBD ante-mortem is still limited.

Clinical core features of PSP are akinetic-rigid syndrome, postural instability or falls and supranuclear ophthalmoplegia (Bensimon et al. 2009). The most recent version of PSP criteria published by the Movement Disorder Society includes eleven clinical phenotypes of PSP (Ali and Josephs 2018). Overall, the clinical diagnoses of post-mortem-validated PSP cases were correctly established only in 19% of cases at the first clinical visit, and in 71% of cases over the course of the disease (Respondek et al. 2013). Furthermore, an average of 24% of post-mortem histopathologically diagnosed cases of MSA, PD and CBD were, ante-mortem, falsely diagnosed as PSP (Respondek et al. 2013). Also, the occurrence of concomitant AD (in approximately 36%) or PD (in approximately 20%) pathologies in PSP patients is remarkable (Dugger et al. 2014).

Histopathologic characteristics of MSA are inclusions of misfolded α-synuclein in oligodendrocytes (Jellinger 2014). According to the clinical presentation, MSA is usually subdivided into a parkinsonian subtype (MSA-P) and a cerebellar subtype (MSA-C). For the clinical phenotype, autonomic failure is a core symptom which must be present to establish the diagnosis of MSA (Gilman et al. 2008).

### Lewy body dementia pathologies

Dementia with Lewy-bodies (DLB) is an α-synucleinopathy, characterized by widespread accumulation of Lewy bodies and Lewy neurites in the brain-stem, limbic system and cortical areas (Braak and Braak 2000) and a higher percentage of DLB compared to PD patients with dementia (PDD) show Aβ deposits in histopathological examinations. Consequently, approximately 80% of DLB patients have a positive Aβ PET scan (Drzezga 2010) (Table 1) indicating a significant overlap between AD and DLB, which is reflected in the clinical presentation of the patients. Only 50% of patients with DLB pathology show the typical symptoms of DLB (McKeith et al. 2016). Core clinical features are fluctuating cognition, recurrent visual hallucinations, rapid eye movement (REM) sleep behaviour disorder and parkinsonism (McKeith et al. 2017). However, “the likelihood that the observed neuropathology explains the DLB clinical syndrome is directly related to the severity of Lewy-related pathology, and inversely related to the severity of concurrent AD-type pathology” (McKeith et al. 2005).

More than 75% of PDD patients develop in the long term clinical course of the disease (Aarsland et al. 2003). PDD is characterized by an intracellular accumulation of α-synuclein (Lewy bodies). Approximately 15% of PDD patients show cerebral β-amyloid plaques (Lucero et al. 2015; Edison et al. 2008). Due to an overlap of clinical and morphological features there is a continuous debate (Friedman 2018) of whether DLB and PDD are the same disease with different phenotypic representation of Lewy body disease spectrum.

### Huntington’s disease (HD)

HD is an autosomal-dominant neurodegenerative disease caused by a single gene mutation (Kim and Fung 2014) i.e. a CAG repeat expansion in exon 1 of the huntingtin gene (MacDonald et al. 1993). This repeated CAG expression results in an abnormal toxic protein which is named Huntingtin. This protein is expressed in all brain cells and disturbs protein degradation as well as several other cellular processes, e.g. mitochondrial function, axonal trafficking or peripheral immune regulation (Kim and Fung 2014). The cerebral accumulation of hyperphosphorylated tau aggregates seems to be a further histopathological characteristic besides the accumulation of the mutated Huntingtin protein (Vuono et al. 2015). From the clinical perspective, HD is characterized by progressive motor symptoms, cognitive decline and neuropsychiatric disturbances (Kim and Fung 2014).

## Role of PET imaging in neurodegenerative diseases

Currently, the classification of neurodegenerative diseases is in permanent change and progress. Predominantly, phenotypical definitions are increasingly substituted – at least in research settings- by classifications which include biomarkers for the underlying pathophysiological process and thus lead to a more biological definition of neurodegenerative diseases (Jack et al. 2018).

This development is of great significance for nuclear medicine, as molecular imaging using PET tracers can provide biomarker information, e.g. [^18^F]FDG as a biomarker of neuronal injury or Aβ PET as a biomarker of AD pathology (Barthel et al. 2015). Table 2 summarizes all radiotracers mentioned in the review with abbreviation and chemical definition.

**Table 2:** Chemical structures of all radiotracers discussed

Abbreviation	Chemical Structure
[^11^C]A-582941	2-[^11^C]methyl-5-[6-phenylpyridazine-3-yl]octahydropyrrolo[3,4-c]pyrrole
[^11^C]A-844606	2 (5-[^11^C]methyl-1,3,3a,4,6,6a-hexahydropyrrolo[3,4-c]pyrrol-5-yl]-4a,9a-dihydroxanthen-9-one
[^11^C]AZD2184	2-(6-[^11^C]methylaminopyridin-3-yl)-1,3-benzothiazol-6-ol
[^11^C]CHIBA-1001	(4-[^11^C]methylphenyl)-1,4-diazabicyclo[3.2.2]nonane-4-carboxylate
[^11^C]cocaine	methyl(1R,2R,3S,5S)-3-(benzoyloxy)-8-[^11^C]methyl-8-azabicyclo[3.2.1]octan-2-carboxylat
[^11^C]DAA1106	N-(5-fluoro-2-phenoxyphenyl)-N-[(5-methoxy-2-[^11^C]methoxyphenyl)methyl]acetamide
[^11^C]JNJ7777120	1-[(5-chloro-1H-indol-2-yl)carbonyl]-4-[^11^C]methylpiperazine
[^11^C]KTP-ME	2-(3-benzoyl-phenyl)-propionic acid-[^11^C]methylester
[^11^C]methylphenidate	[^11^C]methylphenyl-piperidin-2-yl-acetic-acid
[^11^C]MP4A	N-[^11^C]methylpiperidin-4-yl acetate
[^11^C]NS14492	4-{5-[1-[^11^C]methyl-1H-pyrrol-2-yl]-1,3,4-oxadiazol-2-yl}-1,4-diazabicyclo[3.2.2]nonane
[^11^C]PBB3	2-[(1E,3E)-4-[6-([^11^C]methylamino)pyridin-3-yl]buta-1,3-dienyl]-1,3-benzothiazol-6-ol
[^11^C]PBR-28	N-[(2-[^11^C]methoxyphenyl)methyl]-N-(6-phenoxypyridin-3-yl)acetamide
[^11^C]PiB	2-[4-([^11^C]methylamino)phenyl]-1,3-benzothiazol-6-ol
[^11^C]PK-11195	N-sec-Butyl-1-(2-chlorophenyl)-N-[^11^C]methyl-3-isoquinolinecarboxamide
[^11^C] PMP	(1-[^11^C]methylpiperidin-4-yl)propionate
[^11^C]raclopride	3,5-dichloro-N-[[(2S)-1-ethylpyrrolidin-2-yl]methyl]-2-hydroxy-6-[^11^C]methoxybenzamide
[^11^C]UCB-J	((R)-1-((3-([^11^C]methylpyridin-4-yl)methyl)-4-(3,4,5-trifluorophenyl)pyrrolidin-2-one)
2-[^18^F]F-A-85380	2-[^18^F]fluoro-3-(2(S)-azetidinylmethoxy)pyridine
6-[^18^F]F-A-85380	6-[^18^F]fluoro-3-(2(S)-azetidinylmethoxy)pyridine
(−)-[^18^F]flubatine	(−)-(1R,5S,6S)-6-(6-[^18^F]fluoro-pyridine-3-yl)-8-aza-bicyclo[3.2.1]octane
(+)-[^18^F]flubatine	(+)-(1S,5R,6R)-6-(6-[^18^F]fluoro-pyridine-3-yl)-8-aza-bicyclo[3.2.1]octane
[^18^F] ASEM	3-(1,4-diazabicyclo[3.2.2]nonan-4-yl)-6-[^18^F] fluoranyldibenzothiophene 5,5-dioxide
[^18^F]AV-133	(2R,3R,11bR)-9-(3-[^18^F]fluoranylpropoxy)-10-methoxy-3-(2-methylpropyl)-2,3,4,6,7,11b-hexahydro-1H-benzo[a]quinolizin-2-ol
[^18^F]AV-1451	7-(6-[^18^F]fluoranylpyridin-3-yl)-5H-pyrido[4,3-b]indole
[^18^F]AZAN	(1R,2R,4S)-2-[5-(6-[^18^F]fluoranylpyridin-2-yl)pyridin-3-yl]-7-methyl-7-azabicyclo[2.2.1]heptane
[^18^F]DBT-10	(7-(1,4-diazabicyclo[3.2.2]nonan-4-yl)-2-([^18^F]fluorodibenzo[b,d]thiophene 5,5-dioxide)
[^18^F]DPA-714	[N,N-diethyl-2-(2-(4-(2[^18^F]fluoroethoxy)phenyl)5,7dimethylpyrazolo[1,5a]pyrimidin-3-yl)acetamide]
[^18^F]FDOPA	(2S)-2-amino-3-(2-[^18^F]fluoranyl-4,5-dihydroxyphenyl)propanoic acid
[^18^F]FDG	(2S,3R,4S,5S,6R)-3-[^18^F]fluoranyl-6-(hydroxymethyl)oxane-2,4,5-triol
[^18^F]FE-PE2I	(E)-N-(3-iodoprop-2-enyl)-2β-carbo[^18^F]fluoroethoxy-3β-(4′-methyl-phenyl)nortropane
[^18^F]FEPPA	N-[[2-(2-[^18^F]fluoranylethoxy)phenyl]methyl]-N-(4-phenoxypyridin-3-yl)acetamide
[^18^F]FIBT	2-(p-methylaminophenyl)-7-(2-[^18^F]fluoroethoxy)imidazo-[2,1-b]benzothiazole
[^18^F]florbetaben	4-[(E)-2-[4-[2-[2-(2-[^18^F]fluoranylethoxy)ethoxy]ethoxy]phenyl]ethenyl]-N-methylaniline
[^18^F]florbetapir	4-[(E)-2-[6-[2-[2-(2-[^18^F]fluoranylethoxy)ethoxy]ethoxy]pyridin-3-yl]ethenyl]-N-methylaniline
[^18^F]flutemetamol	2-[3-[^18^F]fluoranyl-4-(methylamino)phenyl]-1,3-benzothiazol-6-ol
[^18^F]GTP1	3-[4-(2-[^18^F]fluoro, 2,2-deuteroethyl)-piperidin-1-yl]-benzo[4,5]imidazo[1,2-a]pyridine
[^18^F]MK-6240	6-[^18^F]fluoranyl-3-pyrrolo[2,3-c]pyridin-1-ylisoquinolin-5-amine
[^18^F]MNI-1126	4-(3,5-di[^18^F]fluoro-phenyl)1-(3-methyl-pyridin-4-ylmethyl)pyrrolidin-2-one
[^18^F]NAV4694	2-[2-[^18^F]fluoro-6-(methylamino)-3-pyridinyl]-1-benzofuran-5-ol
[^18^F]NIDA522131	6-chloro-3-((2-(S)-azetidinyl)methoxy)-5-(2-[^18^F]fluoropyridin-4-yl)pyridine
[^18^F]nifene	3-[[(2S)-2,5-dihydro-1H-pyrrol-2-yl]methoxy]-2-[^18^F]fluoranylpyridine
[^18^F]nifrolene	3-[[(2S)-2,5-dihydro-1H-pyrrol-2-yl]methoxy]-5-(3-[^18^F]fluoranylpropyl)pyridine
[^18^F]nifzetidine	3-(2-(S)-azetidinylmethoxy)-5-(3′-[^18^F]fluoropropyl) pyridine
[^18^F]NS10743	2-(1,4-diazabicyclo[3.2.2]nonan-4-yl)-5-(4-[^18^F]fluoranylphenyl)-1,3,4-oxadiazole
[^18^F]PBR06	N-[(2,5-dimethoxyphenyl)methyl]-2-[^18^F]fluoranyl-N-(2-phenoxyphenyl)acetamide
[^18^F]PBR111	2-(6-Chloro-2-(4-(3-[^18^F]fluoropropoxy)phenyl)imidazo[1,2-a]pyridin-3-yl)-N,N-diethylacetamide
[^18^F]PI-2620	2-(2-[^18^F]fluoro-pyridin-4-yl)-8a,9-dihydro-4bH-1,6,9-triaza-fluorene
[^18^F]RO6958948	2-(6-[^18^F]fluoro-pyridin-3-yl)-9H-1,6,9-triaza-fluorene
[^18^F]THK5351	(2S)-1-[^18^F]fluoranyl-3-[2-[6-(methylamino)pyridin-3-yl]quinolin-6-yl]oxypropan-2-ol
[^18^F]XTRA	2-{5-[2-[^18^F]fluoropyridin-4-yl]pyridin-3-yl]-7-methyl-7-azabicyclo[2.2.1]heptane
[^18^F]ZW-104	5-(6-[^18^F]fluorohexyn-1-yl)-3-[2(S)-2-azetidinylmethoxy]pyridine
5-[^123^I]I-A-85380	5-[^123^I]iodo-3-(2(S)-azetidinylmethoxy)pyridine
[^123^I]β-CIT	2β-carbomethoxy-3β-(4-[^123^I]iodophenyl)tropane
[^123^I]FP-CIT	methyl(1R,2S,3S,5S)-8-(3-fluoropropyl)-3-(4-[^123^I]iodanylphenyl)-8-azabicyclo[3.2.1]octane-2-carboxylate
[^123^I]IPT	N-(3-[^123^I]iodopropen-2-yl)-2β-carbomethoxy-3β-(chlorophenyl)tropane
[^123^I]IBVM	5-[^123^I]iodo-3-(4-phenyl-piperidin-1-yl)-1,2,3,4-tetrahydro-naphthalen-2-ol
[^123^I]IBZM	(S-)-2-hydroxy-3-[^123^I]iodo-6-methoxy-N[(1-ethyl-2-pyrrolidinyl)methyl]-benzamide

### Molecular imaging of neurodegeneration

#### [^18^F]FDG PET

**Fig. 1:**
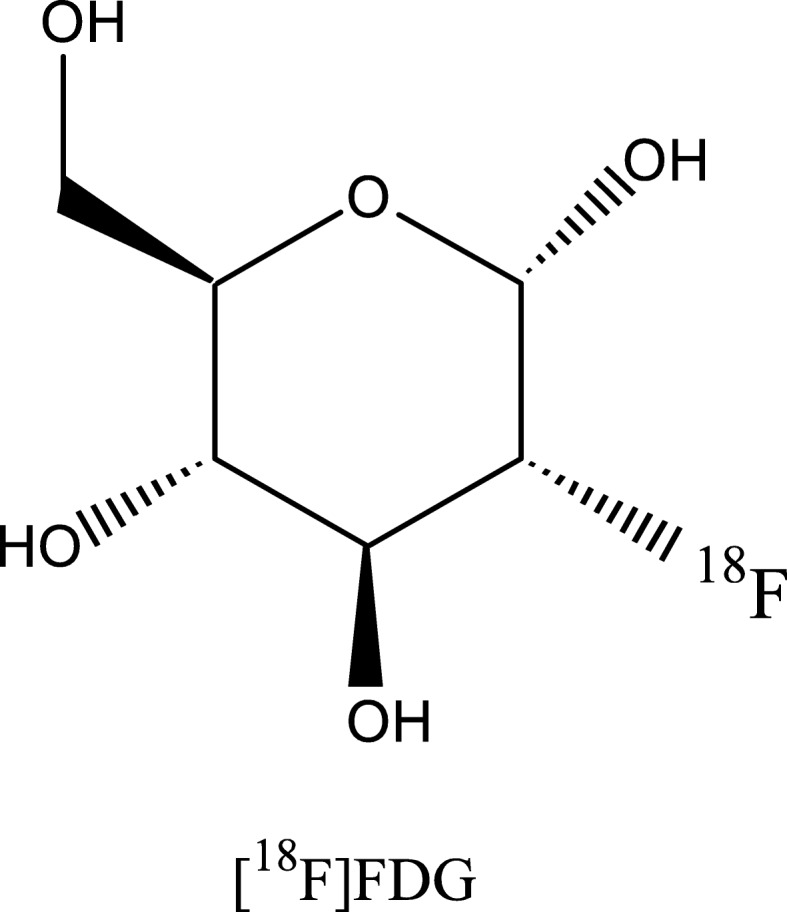
Chemical structure of [^18^F]Fluordesoxyglucose ([^18^F]FDG)

Glucose is the energy supplier of the brain. In all neurodegenerative diseases, impairment of neuronal function and therefore reduced energy metabolism occur. [^18^F]FDG (Fig. 1) as a marker for neuronal injury can be used to detect this impairment, and it is well known that different neurodegenerative diseases show distinct patterns of reduced [^18^F]FDG uptake (Hellwig et al. 2012; Barthel et al. 2015) (Table 3). However, the hypometabolic patterns of some neurodegenerative disorders overlap. Due to its broad availability and sufficient diagnostic accuracy, [^18^F]FDG is currently the widely used radiotracer in imaging of neurodegenerative diseases in clinical routine.

**Table 3:** Summary of typical hypometabolism patterns in [^18^F] FDG PET in different neurodegenerative diseases

Disease	Relative glucose metabolism reduction	Disease	Relative glucose metabolism reduction
AD	PCC, parieto-temporal*. Advanced*: frontal	PD/DLB	Parieto-temporo-occiptal^a^
PCA	Parieto-occipital	PSP	Mesial and dorsolateral, caudate, thalamus, upper brain stem^a^
lvPPA	Parieto-temporal (left pronounced)	CBS	Fronto-parietal, striatal (asymmetric)^a^
bvFTD	Frontal, ACC, right anterior insula. *Advanced:* temporal and subcortical	MSA	Striatum (posterior putamen), cerebellum^a^
svPPA	Anterior temporal, subcallosal, amygdalae, frontal midline	HD	Striatum, insula, posterior cingulate, prefrontal, occipital cortex^b^
nfvPPA	Left hemisphere, frontotemporal, insula. *Advanced:* parieto-temporal		

*ACC* Anterior cingulate cortex, *AD* Alzheimer’s disease, *bvFTD* Behavioural variant frontotemporal dementia, *CBS* Corticobasal syndrome, *DLB* Dementia with Lewy-bodies, *FDG* Fluorodesoxyglucose, *HD* Huntington’s disease, *lvPPA* Logopenic variant primary progressive aphasia, *MSA* Multisystem atrophy, *nfvPPA* non-fluent/agrammatic variant primary progressive aphasia, *PCA* Posterior cortical atrophy, *PCC* Posterior cingulate cortex, *PD* Parkinson’s disease, *PET* Positron emission tomography, *PSP* Progressive supranuclear palsy, *svPPA* Semantic variant primary progressive aphasia

^a^(Meyer et al. 2017) ^b^(Tang et al. 2013)

#### Synaptic density PET

**Fig. 2:**
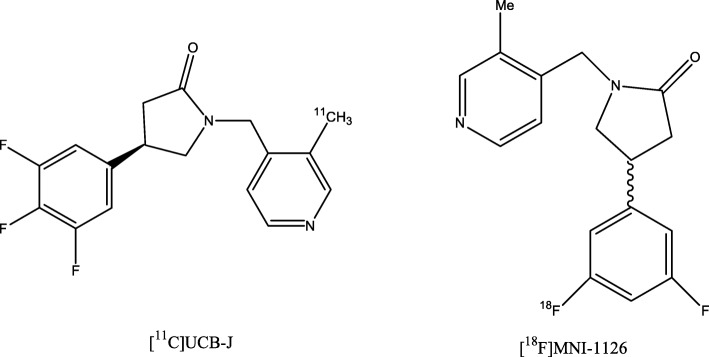
Chemical structure of ((R)-1-((3-([^11^C]-methyl-[^11^C])pyridin-4-yl)methyl)-4-(3,4,5-trifluorophenyl)pyrrolidin-2-one) ([^11^C]UCB-J) and its F18-labeled radioligand derivative *(R)-*[^18^F]MNI-116

In neurodegenerative diseases as well as in a variety of other neurological and psychiatric diseases, a reduction of synaptic density occurs during the course of disease (Feng et al. 2009; van Vliet et al. 2009; DeKosky and Scheff 1990; Hamos et al. 1989; Kang et al. 2012; Glantz and Lewis 2000). Quantification of synaptic density is usually performed post-mortem. Development of levetiracetam-based PET radioligands targeting synaptic vesicle glycoprotein 2A (SVA2) now enables the in-vivo quantification of this parameter (Koole et al. 2018; Finnema et al. 2016). The most recently developed tracer [^11^C]UCB-J (Fig. 2) has demonstrated favorable pharmacokinetics and quantification properties in preclinical as well as in first-in-human studies (Finnema et al. 2016). An ^18^F-labeled derivative ([^18^F]MNI-1126) (Fig. 2) has also been evaluated recently in non-human primates showing promising in-vivo characteristics (Constantinescu et al. 2018).

### Imaging of neuroinflammation

**Fig. 3:**
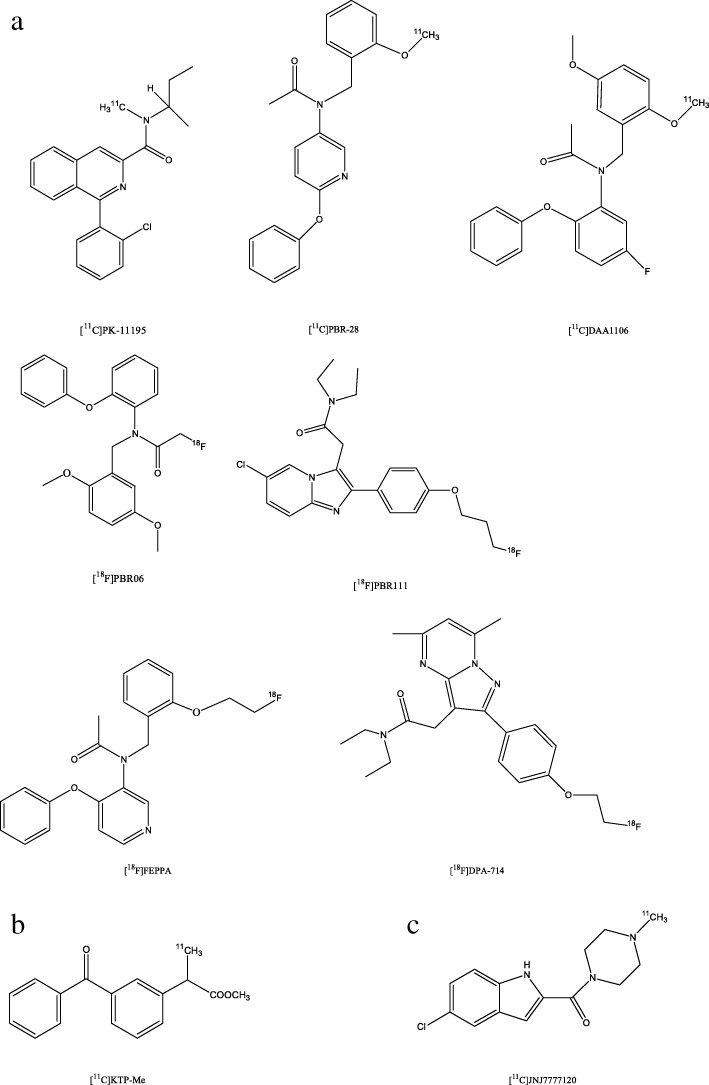
Chemical structures of PET radioligands for imaging neuroinflammation – targeting: **a** TSPO, **b** cyclooxygenase 1 and **c** histamine 4 receptor

Many neurodegenerative diseases are also accompanied, if not - as some researchers believe (Krstic and Knuesel 2013) - caused by inflammatory processes which are mainly mediated by activated microglia. The 18 kDa translocator protein (TSPO) is upregulated in glial cells during inflammation (Albrecht et al. 2016) and, as such, a target for PET neuroimaging. [^11^C]PK-11195 (Fig. 3a) is the most-studied TSPO radiotracer. However, it has a low brain penetrance and a high non-specific binding resulting in a poor signal-to-noise ratio (Albrecht et al. 2016; Ory et al. 2014). The second-generation TSPO radiotracers (e.g. [^11^C]PBR-28, [^18^F]DPA-714, [^18^F] FEPPA, [^11^C]DAA1106, [^18^F]PBR06, [^18^F]PBR111) (Fig. 3a) have several advantages as compared to [^11^C]PK-11195, like a higher signal-to-background-ratio. As a potential drawback, however, the interpretation of their uptake is confounded by the existence of three different binding affinities (low-affinity, high-affinity, and mixed-affinity binder) (Albrecht et al. 2016; Herrera-Rivero et al. 2015). Other targets for an indirect measure of neuroinflammation have been identified, e.g. cyclooxygenase 1, histamine 4 receptors, alpha7-nicotinic acetylcholine receptors, and others (Ory et al. 2014; Albrecht et al. 2016). Several PET tracers for these targets have been developed and optimized e.g., [^11^C]KTP-ME (Fig. 3b) for imaging cyclooxygenase 1 and [^11^C]JNJ7777120 (Fig. 3c) for imaging histamine 4 receptors (Ory et al. 2014; Albrecht et al. 2016). However, neuroinflammation is a highly complex process which is not fully understood and more recently published data on PET imaging of cyclooxygenase 1 or histamine 4 receptors are missing. Radiotracers targeting alpha7-nAChRs are described in below in the *Cholinergic System Imaging* section*.*

### Imaging of neurotransmission

#### Dopaminergic system imaging

**Fig. 4:**
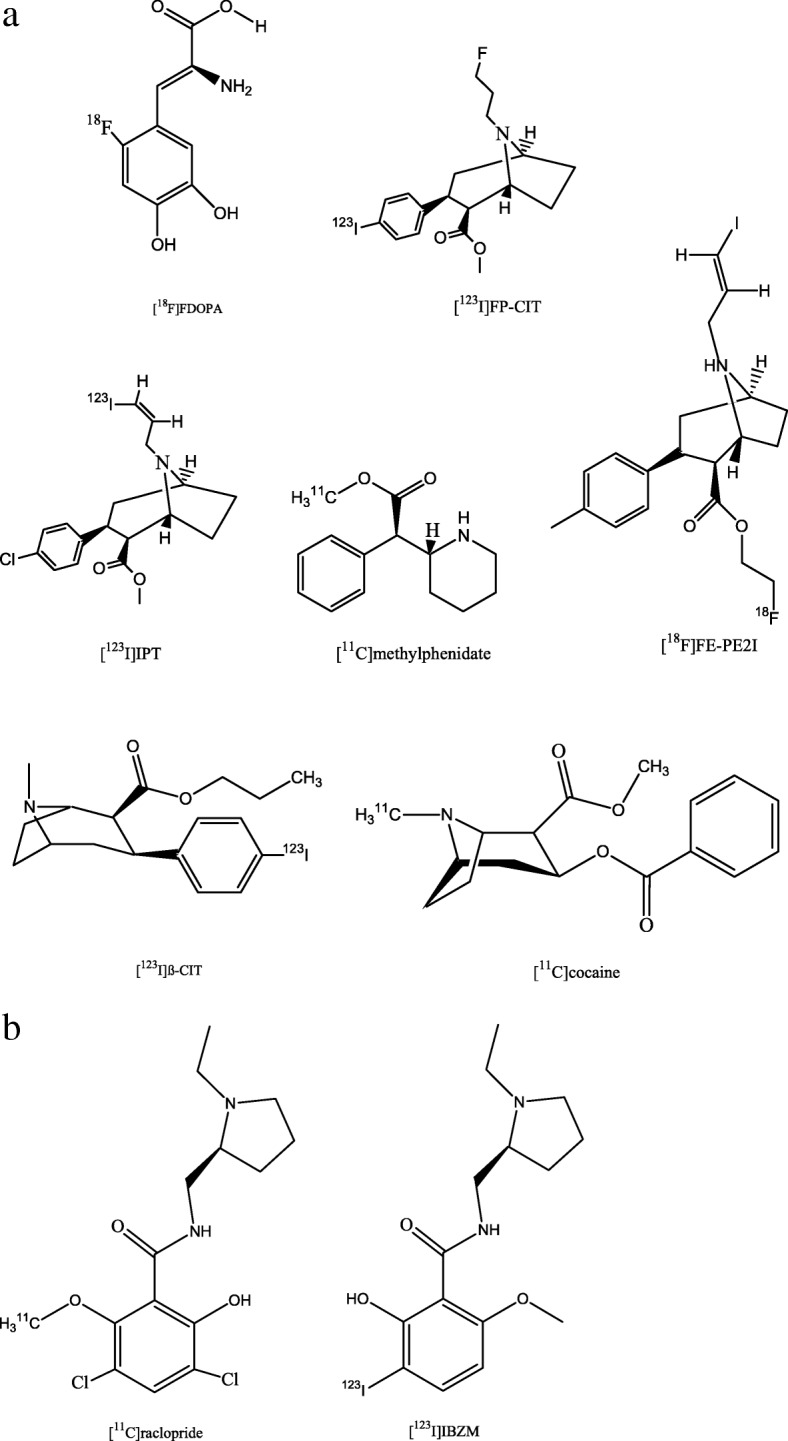
Chemical structures of radiotracers for imaging the dopaminergic system –targeting: **a** presynaptic structures and **b** postsynaptic receptors

Dopamine is a neurotransmitter involved in movement, cognition, motivation and addiction. Because the dopaminergic system is crucially involved in the pathophysiology of parkinsonism, imaging of the dopaminergic system is of particular interest in diseases such as Parkinson’s disease or variants of atypical parkinsonism. The PET tracer [^18^F]FDOPA (Fig. 4a) is a structural analogue of L-DOPA which is a precursor of dopamine. By measuring the uptake of dopamine precursors, [^18^F]FDOPA can be used to investigate the integrity of the dopaminergic system (Leenders et al. 1990). Another approach to detecting the integrity of dopaminergic neurons is imaging of presynaptic membrane DAT using SPECT tracer tropane derivatives (i.e. [^123^I]β-CIT, [^123^I]FP-CIT (DaTSCAN™), [^123^I]IPT) and PET tracers [^11^C]methylphenidate, [^11^C]Cocaine, or [^18^F]FE-PE2I (Seibyl 2008) (Fig. 4a).

Most neurodegenerative Parkinsonian syndromes such as idiopathic PD, atypical PD and DLB are associated, in contrast to drug-induced parkinsonism or essential tremor, with a loss of presynaptic dopaminergic neurons. Thus, imaging of the integrity of presynaptic dopaminergic function enables to differentiate neurodegenerative Parkinsonian syndromes from essential tremor or drug-induced parkinsonism (Seibyl 2008). Further, imaging of the integrity of presynaptic dopaminergic function is useful to differentiate AD from DLB (Minoshima et al. 2004).

Imaging of postsynaptic D_2/3_ receptors with selective radioligands such as [^11^C]raclopride (for PET) and [^123^I]IBZM (for SPECT) (Fig. 4b) was used for a longer time to differentiate PD from atypical (i.e. PSP, MSA, CBD, DLB) parkinsonism. Recent research, however, demonstrated that [^18^F]FDG PET is superior to [^123^I]IBZM SPECT in this regard. This is as [^18^F]FDG PET allows not only to discriminate specific variants of atypical parkinsonism with high accuracy (Hellwig et al. 2012).

#### Cholinergic system imaging

**Fig. 5:**
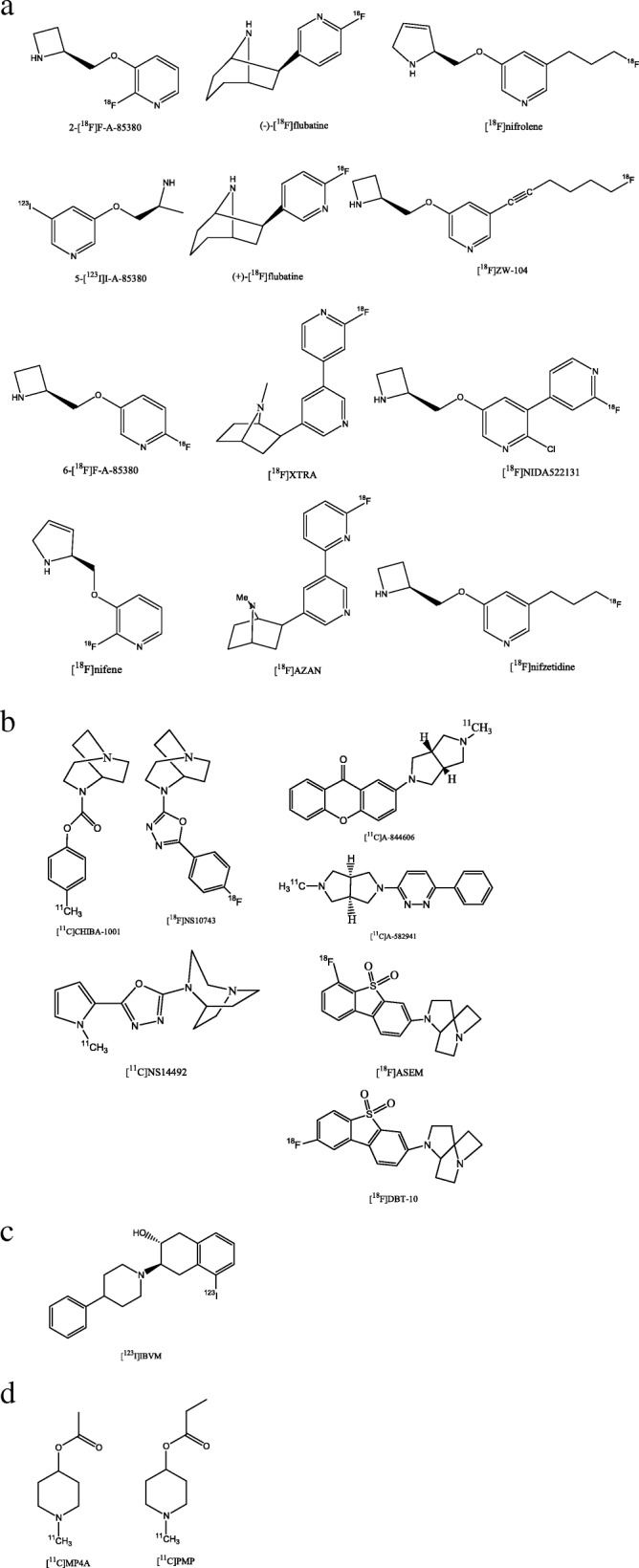
Chemical structures of radiotracers for imaging the cholinergic system –targeting: **a** α4β2 nicotinic acetylcholine receptors (nAChRs), **b** α7 nAChRs, **c** vesicular acetylcholine transporter and **d** acetylcholinesterase

Autoradiographic data revealed a significant reduction of different compartments of cholinergic neurotransmission, like nicotinic acetylcholine receptors (nAChRs) in patients with AD, PD and DLB (Perry et al. 1995; Martin-Ruiz et al. 2000; Flynn and Mash 1986; Sihver et al. 1999). These data are in support of the cholinergic hypothesis of geriatric memory dysfunction which assumes that cognitive declines are mainly caused by a reduction of acetylcholine in the synaptic cleft as a consequence of a reduction of nicotinic neurons (Bartus et al. 1982). In autoradiographic studies, reductions of the α4 subunit of the nAChR were detected in the range of 50–65% in moderate-severe stage AD (Sihver et al. 1999; Martin-Ruiz et al. 2000) and 30–50% in moderate stage DLB (Martin-Ruiz et al. 2000). In contrast, in-vivo PET/SPECT studies using α4β2 nAChR-targeting radioligands demonstrated reductions to a more variable degree. This can be at least partly explained by the fact that distinct methods for quantification (e.g. binding potentials, distribution volumes, distribution volume ratios) of α4β2 nAChR availability were used and by the fact that patients cohorts differed between studies in severity of disease ranging from mild to moderate stage of the disease (O'Brien et al. 2007; Sabri et al. 2008; Meyer et al. 2009; Kendziorra et al. 2011; Meyer et al. 2014; Sultzer et al. 2017; Sabri et al. 2018). Most important and widely used early-generation α4β2 nAChR PET ligands are 3-pyridylether derivatives such as 2-[^18^F]A-85380, 6-[^18^F]A-85380 and 5-[^123^I]A-85380 (Fig. 5a). As these radioligands exhibit slow kinetics resulting in long scanning times, new radioligands with more favourable characteristics have been developed and tested in preclinical and first clinical trials (Horti et al. 2013). These next-generation α4β2 nAChR radioligands are derivatives of homoepibatidine ((−)-[^18^F]Flubatine, (+)-[^18^F]Flubatine), epibatidine ([^18^F]AZAN, [^18^F]XTRA) or 3-pyridylether derivatives ([^18^F]Nifene, [^18^F]Nifrolene and [^18^F]NIDA522131, [^18^F]Nifzetidine and [^18^F]ZW-104) (Horti et al. 2013; Meyer et al. 2014) (Fig. 5a). Results of the first applications in humans of these next-generation α4β2 nAChR-targeting PET radioligands such as (−)-[^18^F]Flubatine, [^18^F]AZAN and [^18^F]XTRA are promising (Sabri et al. 2015a, 2015b; Sabri et al. 2018; Wong et al. 2013; Coughlin et al. 2018a, 2018b). Especially, the faster kinetics with sufficient estimation of radiotracer binding within 90 min (Sabri et al. 2015a, 2015b; Wong et al. 2013; Coughlin et al. 2018a, 2018b) is an advantage compared to the early-generation α4β2 nAChR PET ligands. Further advantages are: (a) for (−)-[^18^F]Flubatine a small metabolization which allows quantification without metabolite correction (Sabri et al. 2015a, 2015b), (b) for [^18^F]AZAN a greater specific binding compared to 2-[^18^F]A-85380 (Wong et al. 2013) and (c) for [^18^F]XTRA higher distribution volumes in extrathalamic brain regions compared to the published data of [^18^F]AZAN and (−)-[^18^F]Flubatine, whereby a displacement study needs to clarify whether these higher distribution volumes are due to specific or nonspecific binding (Coughlin et al. 2018a, 2018b).

In addition to the α4β2 subtype, also α7 nAChRs should play an important role in the pathophysiologic processes, for instance, in AD (Bao et al. 2017). α7 nAChRs seem to mediate Aβ-induced tau protein hyperphosphorylation (Wang et al. 2003) and modulate immunological process in AD (Conejero-Goldberg et al. 2008) and probably, in other neurodegenerative diseases. First α7 nAChR-targeting PET radioligands i.e. [^11^C]CHIBA-1001 and [^18^F]ASEM (Fig. 5b) have been evaluated in humans (Ishikawa et al. 2011; Wong et al. 2014; Coughlin et al. 2018a, 2018b) and other promising radioligands e.g. [^11^C]A-582941 and [^11^C]A-844606 (Toyohara et al. 2010), [^18^F]NS10743 (Deuther-Conrad et al. 2011) [^11^C]NS14492 (Ettrup et al. 2011), [^18^F]DBT-10 (Hillmer et al. 2016) have been preclinically examined (Fig. 5b).

Apart from nAChR deficiency, post-mortem data revealed reductions of vesicular acetylcholine transporter (VChAT) and acetylcholinesterase (AChE) in AD patients compared to healthy controls (HCs) and, further, a correlation between neocortical AChE activity and dementia severity (Bierer et al. 1995). Therefore, ante-mortem examination of VChAT and AChE activity could be also of interest in AD. A radioligand targeting VChAT is [^123^I]IBVM (Fig. 5c) and radioligands targeting AChE are [^11^C]MP4A and [^11^C]PMP (Kuhl et al. 1996; Kuhl et al. 1999; Roy et al. 2016) (Fig. 5d). Furthermore, [^18^F]FEOBV, a novel, very promising PET radioligand targeting VChAT has been developed and successfully applied in patients with AD and PD (Aghourian et al. 2017; Bohnen et al. 2019). Thus, using abovementioned radioligands, reduced activities of VChAT and AChE were demonstrated in various neurodegenerative diseases like AD, PD and DLB (Kuhl et al. 1996; Kuhl et al. 1999; Roy et al. 2016; Aghourian et al. 2017; Bohnen et al. 2019).

#### Monoamine system imaging

**Fig. 6:**
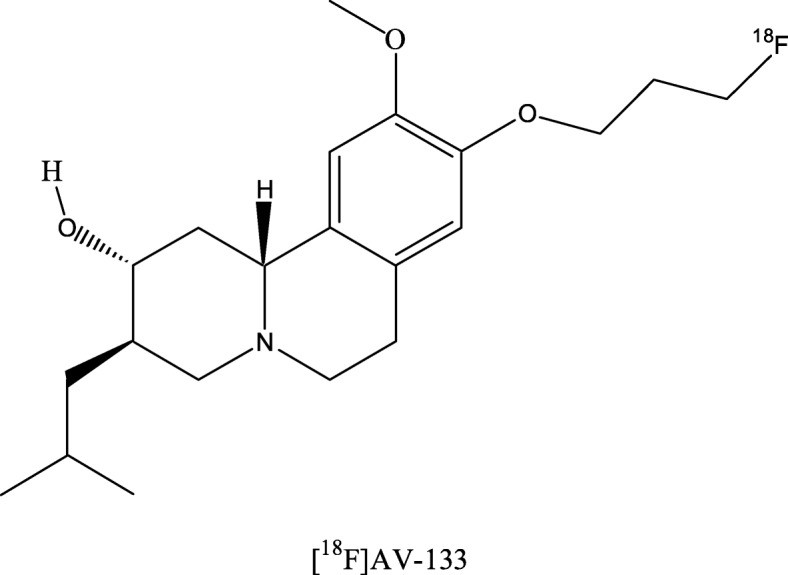
Chemical structure of (2R,3R,11bR)-9-(3-[^18^F]fluoranylpropoxy)-10-methoxy-3-(2-methylpropyl)-2,3,4,6,7,11b-hexahydro-1H-benzo [a]quinolizin-2-ol ([^18^F]AV-133)

The vesicular monoamine transporter 2 (VMAT2) is a membrane protein that transports monoamines (e.g. dopamine or serotonin) into the presynaptic vesicles. [^18^F]AV-133 (Fig. 6) is a PET radiotracer targeting VMAT2. In patients with PD, a reduced [^18^F]AV-133 uptake was found in the basal ganglia, more pronounced in the putamen and contralateral to the predominantly affected side at onset (Gao et al. 2016). An accuracy in differentiating PD patients from HCs similar to that of DAT SPECT has been reported. Furthermore, [^18^F]AV-133 PET data might better correlate to clinical characteristics than PET/SPECT imaging data of DAT (Hsiao et al. 2014).

### Imaging of misfolded proteins

#### β-Amyloid (Aβ) PET imaging

**Fig. 7:**
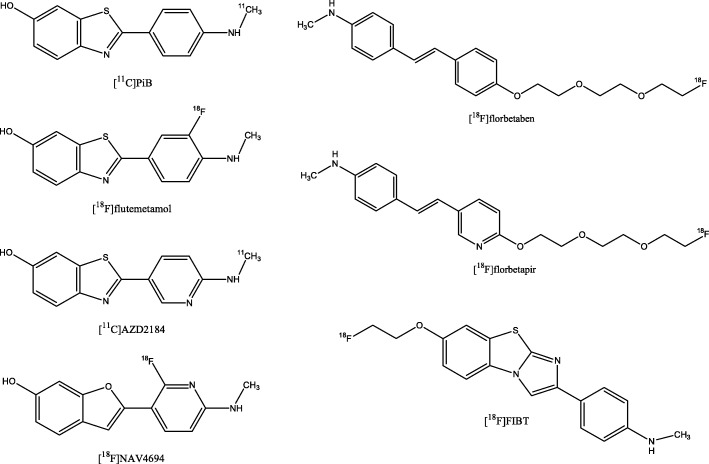
Chemical structures of the β-amyloid targeting PET radioligands

Aβ plaques are the histopathological hallmark of AD. Moreover, their appearance in the brain is an early, if not the causal event in AD. The most widely used Aβ-targeting PET tracer is [^11^C]Pittsburgh Compound B (PiB) (Fig. 7). However, the short half-life hampers the use of this tracer for clinical routine applications. Thus, three ^18^F-labeled radiotracers (i.e. florbetapir, florbetaben, flutemetamol) Fig. 7) have been developed and approved for clinical usage. The phase 3 data of all 3 radiotracers demonstrated high sensitivity ([^18^F]florbetapir: 96%, [^18^F]florbetaben: 98%, [^18^F]flutemetamol: 88%) and specificity ([^18^F]florbetapir: 100%, [^18^F]florbetaben: 89%, [^18^F]flutemetamol: > 80%) in detecting Aβ plaques in-vivo compared to the postmortem data (Clark et al. 2012; Sabri et al. 2015a, 2015b; Curtis et al. 2015). Other Aβ PET tracers, such as [^11^C]AZD2184, [^18^F]FIBT, and [^18^F]NAV4694 (Fig. 7), are under clinical examination (Ito et al. 2014; Grimmer et al. 2018). Results of the first in humans studies revealed a fast kinetics of [^11^C]AZD2184 and [^18^F] FIBT, and a time-window of 40–60 min p.i. was determined as reliable to calculate standard uptake value ratios (SUVRs) (Ito et al. 2014; Grimmer et al. 2018). The kinetics is therefore comparable to that of [^18^F]florbetapir and [^18^F]florbetaben where an acquisition start 30 min p.i. ([^18^F]florbetapir) and 45 min p.i. for the USA/90 min p.i. for Europe ([^18^F]florbetaben) is recommended (https://eanm.org/publications/guidelines/Amyloid-Guideline-J_Nucl_Med-2016-Minoshima-1316-22.pdf). Compared to the three approved ^18^F-labeled radiotracers, [^11^C]AZD2184 and [^18^F]NAV4694 seem to show lower white matter binding (Ito et al. 2014; Rowe et al. 2013) which principally might translate to a higher sensitivity in detecting subtle amyloid pathology.

Over the last few years, the clinical and research diagnostic criteria especially for AD (McKhann et al. 2011; Albert et al. 2011; Dubois et al. 2014; McKeith et al. 2017; Jack et al. 2018) but also for other neurodegenerative diseases have been revised (McKeith et al. 2017; Berg et al. 2013), resulting in an implementation of biomarkers such as Aβ PET or [^18^F]FDG PET. However, the validation process of these biomarkers is still incomplete (Frisoni et al. 2017). Especially, clinical outcome and cost-effectiveness studies are still missing (Frisoni et al. 2017). As such studies are the decisive prerequisite for reimbursement within many healthcare systems, these PET imaging biomarkers are so far not regularly applied in clinical routine.

#### Tau PET imaging

**Fig. 8:**
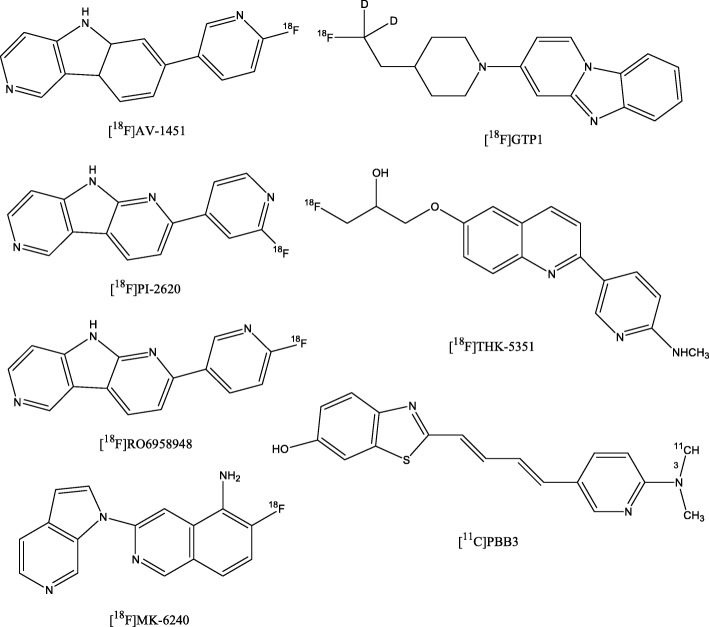
Chemical structures of the Tau targeting PET radioligands

Physiological tau is a phosphoprotein which stabilizes the microtubules. In the brain, six isoforms of tau exist with either three repeats (3R) or four repeats (4R) of the microtubules-binding domain (Buée et al. 2000). Aggregated tau proteins consist of post-translationally modified tau isoforms, whereby specific phenotypes/neurodegenerative diseases are associated with specific tau deposits that differ in microscopic appearance and ultrastructure (Buée et al. 2000; Villemagne et al. 2015). Importantly, the same clinical tauopathy phenotype can be caused by different misfolded tau proteins and vice-versa (Villemagne et al. 2015). In general, aggregated tau proteins are mainly located intracellularly and therefore a complex target for PET imaging. Current tau radiotracers share β-sheet binding properties. Since other misfolded proteins have similar structures, high selectivity for aggregated tau proteins is necessary (Lois et al. 2018). This is of particular interest, as tau aggregates can be co-localized to Aβ plaques with much higher concentrations of Aβ plaques compared to tau deposits (Villemagne et al. 2015). First-generation tau PET radiotracers – [^18^F]AV-1451, [^11^C]PBB3, [^18^F]THK5351 (Fig. 8) – showed favourable kinetics and high affinity to the 3R/4R tau isoform combination which is typical in AD (Villemagne et al. 2015; Lois et al. 2018; Villemagne 2018). However, the limitation of the first-generation tau PET radiotracers are a relevant off-target binding as well as ante-mortem vs. post-mortem inconsistencies (Villemagne et al. 2015; Harada et al. 2018; Lois et al. 2018; Villemagne 2018). Second-generation selective tau PET radiotracers, such as [^18^F]RO6958948, [^18^F]GTP1, [^18^F]PI-2620, [^18^F]MK-6240 (Fig. 8), have been preclinically evaluated and demonstrated high affinity, selectivity and specificity (Lois et al. 2018). Preliminary clinical data, partially available as conference abstracts, are promising (Mueller et al. 2017; Barret et al. 2017; Bohorquez et al. 2016; Wong et al. 2018; Bohorquez et al. 2017; Betthauser et al. 2018). However, [^18^F]GTP1 showed off-target binding in the basal ganglia (Bohorquez et al. 2017), while [^18^F]RO6958948 did so in the substantia nigra (Wong et al. 2018). So far, for [^18^F]MK-6240 and [^18^F]PI-2620 off-target binding was not observed (Betthauser et al. 2018; Barret et al. 2017). Noteworthy, preliminary data also suggest that [^18^F]PI-2620 might not only be able to visualize the 3R/4R tau isoform combination in AD, but also the 4R isoform in 4R-tauopathies such as PSP/CBD (https://www.alzforum.org/news/conference-coverage/next-generation-tau-pet-tracers-strut-their-stuff). Although the available data on the second-generation tau PET radiotracers are encouraging, the usefulness of these radiotracers for research and clinical approaches remains to be demonstrated in larger clinical trials.

#### Imaging of other misfolded proteins

Following the recent success with bringing amyloid and tau PET tracers into humans, the desire for radioligands targeting other misfolded proteins like α-synuclein or TDP-43 is evident. However, developing PET radiotracers that target misfolded proteins beyond Aβ is challenging as these proteins (i) are mainly intracellularly localized, (ii) appear in a much lower concentration than Aβ plaques (Villemagne et al. 2015; Lois et al. 2018; Harada et al. 2018; Verdurand et al. 2018), and (iii) have β-sheet binding motives which are rather similar to those of amyloid aggregates. Despite intensive efforts to develop α-synuclein- and TDP-43-targeting PET radiotracers, until now no suitable substance has been described (Mathis et al. 2017).

The FET protein family consists of fused in sarcoma (FUS), Ewing sarcoma (EWS) and TATA-binding protein associated factor 15 (TAF15) and was first discovered as components of fusion oncogenes causing specific malignancies (Mackenzie and Neumann 2016). As DNA/RNA binding proteins, they are predominantly located in the cell nucleus and are involved in DNA/RNA metabolism as well as in the maintenance of genomic stability (Mackenzie and Neumann 2016; Svetoni et al. 2016). In approximately 5–10% of all FTLD cases, the intracellular inclusions are FTLD-Tau- and TDP-43-negative in immunohistochemical examination. But they can be labeled using FUS/EWS/TAF15 antibodies and are therefore classified as FTLD-FUS or FTLD-FET group (Mackenzie and Neumann 2016). Similar to α-synuclein and TDP-43, the existing literature does not reveal any reports regarding FTLD-FET targeting radiotracers. Considering the low prevalence of these diseases (FTLD-TDP is rare, FTLD-FET is even rarer), the search for suitable radiotracers targeting these proteins is so far less active.

## Summary and conclusion

**Fig. 9:**
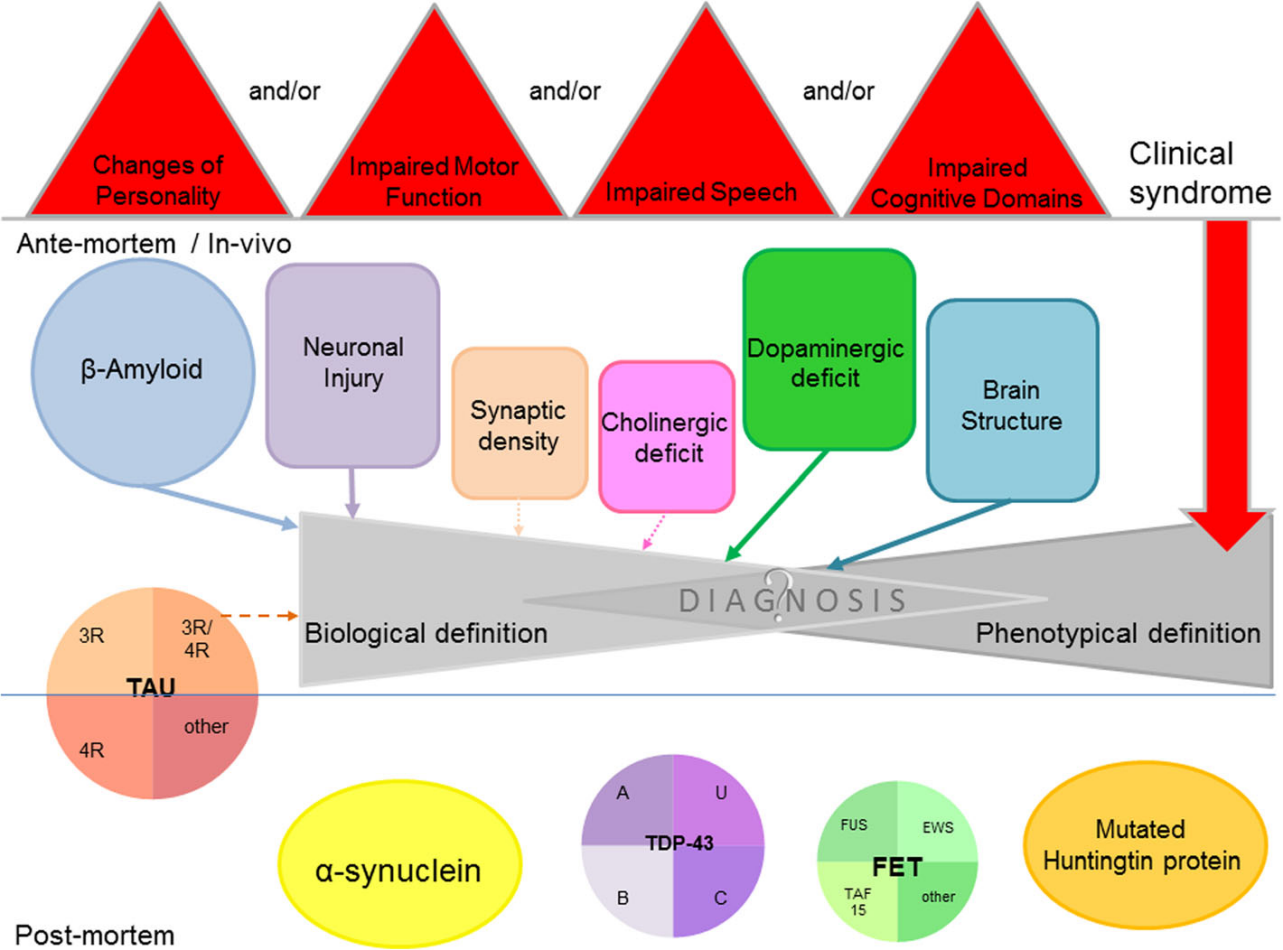
Overview of available imaging biomarkers detecting characteristics of neurodegenerative diseases ante-mortem/in-vivo and histopathological hallmarks which are/were, so far, only detectable post-mortem. Furthermore, the shift from a phenotypical to a biological definition of neurodegenerative diseases as it currently emerges in research settings is addressed

In the last two to three decades, a large number of novel radiotracers for direct and indirect imaging neurodegenerative processes and their underlying pathology have been developed. Several of them have been approved and are used in clinical routine for early and differential diagnosis as well as for evaluation of disease progression. Others are appreciated as valuable research tracers. However, the more pathological components of the different neurodegenerative diseases are discovered, the more new and interesting issues occur. Such issues are (i) the classification of neurodegenerative disorders in clinical routine, (ii) the identification of targets for possible new radiotracers, (iii) the identification of novel radiotracer targets, (iv) the accurate monitoring strategy of such therapy trials. Some of them could be answered by PET studies with new radiotracers. But as important as the development of new radiotracers seems to be, at the moment it is equally important to sum up our gathered pieces of knowledge, combine them and try to get a more comprehensive understanding of the entire spectrum of neurodegenerative disorders (Fig. 9).

## Data Availability

Not applicable.

## Abbreviations

3R: Three repeat tau isoform
4R: Four repeat tau isoform
AChE: Acetylcholine esterase
AD: Alzheimer’s disease
Aβ: β-amyloid
bvFTD: Behavioural variant of frontotemporal dementia
CBD: Corticobasal degeneration
DAT: Dopamine transporter
DLB: Dementia with Lewy-bodies
EWS: Ewing sarcoma
FET: Fused in sarcoma, Ewing sarcoma, TATA-binding protein associated factor 15
FTLD: Frontotemporal lobar degeneration
FUS: Fused in sarcoma
HD: Huntington’s disease
lvPPA: Logopenic variant primary progressive aphasia
MSA: Multisystem atrophy
nAChR: Nicotinic acetylcholine receptors
nfvPPA: Non-fluent/agrammatic variant primary progressive aphasia
PCA: Posterior cortical atrophy
PD: Parkinson’s disease
PDD: Parkinson’s disease dementia
PET: Positron emission tomography
PPA: Primary progressive aphasia
PSP: Progressive supranuclear palsy
SPECT: Single photon emission computed tomography
SVA2: Synaptic vesicle glycoprotein 2A
svPPA: Semantic variant primary progressive aphasia
TAF15: TATA-binding protein associated factor 15
Tau: Hyperphosphorylated neurofibrillary tau protein
TDP-43: Transactive response DNA binding protein of about 43 kDa
TSPO: 15 kDa translocator protein
VChAT: Vesicular acetylcholine transporter
VMAT2: Vesicular monoamine transporter 2

## References

[CR1] Aarsland D, Andersen K, Larsen JP, Lolk A, Kragh-Sørensen P (2003). Prevalence and characteristics of dementia in Parkinson disease: an 8-year prospective study. Arch Neurol.

[CR2] Aghourian M, Legault-Denis C, Soucy JP, Rosa-Neto P, Gauthier S, Kostikov A (2017). Quantification of brain cholinergic denervation in Alzheimer's disease using PET imaging with [^18^F]-FEOBV. Mol Psychiatry.

[CR3] Albert MS, DeKosky ST, Dickson D, Dubois B, Feldman HH, Fox NC (2011). The diagnosis of mild cognitive impairment due to Alzheimer's disease: recommendations from the National Institute on Aging-Alzheimer's Association workgroups on diagnostic guidelines for Alzheimer's disease. Alzheimers Dement.

[CR4] Albrecht DS, Granziera C, Hooker JM, Loggia ML (2016). In vivo imaging of human Neuroinflammation. ACS Chem Neurosci.

[CR5] Ali F, Josephs K (2018). The diagnosis of progressive supranuclear palsy: current opinions and challenges. Expert Rev Neurother.

[CR6] Armstrong MJ, Litvan I, Lang AE, Bak TH, Bhatia KP, Borroni B (2013). Criteria for the diagnosis of corticobasal degeneration. Neurology..

[CR7] Bang J, Spina S, Miller BL (2015). Frontotemporal dementia. Lancet..

[CR8] Bao W, Jia H, Finnema S, Cai Z, Carson RE, Huang YH (2017). PET imaging for early detection of Alzheimer's disease: from pathologic to physiologic biomarkers. PET Clin.

[CR9] Barret O, Seibyl J, Stephens A, Madonia J, Alagille D, Mueller A (2017). Initial clinical PET studies with novel Tau agent [^18^F]PI2620 in Alzheimer’s disease and controls. J Nucl Med.

[CR10] Barthel H, Schroeter ML, Hoffmann K-T, Sabri O (2015). PET/MR in dementia and other neurodegenerative diseases. Semin Nucl Med.

[CR11] Bartus RT, Dean RL, Beer B, Lippa AS (1982). The cholinergic hypothesis of geriatric memory dysfunction. Science..

[CR12] Bensimon G, Ludolph A, Agid Y, Vidailhet M, Payan C, Leigh PN (2009). Riluzole treatment, survival and diagnostic criteria in Parkinson plus disorders: the NNIPPS study. Brain..

[CR13] Berg D, Lang AE, Postuma RB, Maetzler W, Deuschl G, Gasser T (2013). Changing the research criteria for the diagnosis of Parkinson's disease: obstacles and opportunities. Lancet Neurol.

[CR14] Betthauser Tobey J., Cody Karly A., Zammit Matthew D., Murali Dhanabalan, Converse Alexander K., Barnhart Todd E., Stone Charles K., Rowley Howard A., Johnson Sterling C., Christian Bradley T. (2018). In Vivo Characterization and Quantification of Neurofibrillary Tau PET Radioligand 18F-MK-6240 in Humans from Alzheimer Disease Dementia to Young Controls. Journal of Nuclear Medicine.

[CR15] Bierer LM, Haroutunian V, Gabriel S, Knott PJ, Carlin LS, Purohit DP (1995). Neurochemical correlates of dementia severity in Alzheimer's disease: relative importance of the cholinergic deficits. J Neurochem.

[CR16] Bohnen Nicolaas I., Kanel Prabesh, Zhou Zhi, Koeppe Robert A., Frey Kirk A., Dauer William T., Albin Roger L., Müller Martijn L.T.M. (2019). Cholinergic system changes of falls and freezing of gait in Parkinson's disease. Annals of Neurology.

[CR17] Bohorquez SS, Barret O, Tamagnan G, Alagille D, Marik J, Ayalon G (2016). Evaluation of tau burden in a cross-sectional cohort of Alzheimer’s disease subjects using [^18^F]GTP1 (Genentech tau probe 1). Alzheimer’s Dement.

[CR18] Bohorquez SS (2017). Kinetics of [^18^F]GTP1 (Genentech tau probe 1) in the basal ganglia of Alzheimer’s patients and healthy controls.

[CR19] Braak H, Braak E (2000). Pathoanatomy of Parkinson's disease. J Neurol.

[CR20] Buée L, Bussière T, Buée-Scherrer V, Delacourte A, Hof PR (2000). Tau protein isoforms, phosphorylation and role in neurodegenerative disorders. Brain Res Brain Res Rev.

[CR21] Caso F, Mandelli ML, Henry M, Gesierich B, Bettcher BM, Ogar J (2014). In vivo signatures of nonfluent/agrammatic primary progressive aphasia caused by FTLD pathology. Neurology..

[CR22] Clark CM, Pontecorvo MJ, Beach TG, Bedell BJ, Coleman RE, Doraiswamy PM (2012). Cerebral PET with florbetapir compared with neuropathology at autopsy for detection of neuritic amyloid-β plaques: a prospective cohort study. Lancet Neurol.

[CR23] Conejero-Goldberg C, Davies P, Ulloa L (2008). Alpha7 nicotinic acetylcholine receptor: a link between inflammation and neurodegeneration. Neurosci Biobehav Rev.

[CR24] Constantinescu Cristian C., Tresse Cedric, Zheng MingQiang, Gouasmat Alexandra, Carroll Vincent M, Mistico Laetitia, Alagille David, Sandiego Christine M., Papin Caroline, Marek Kenneth, Seibyl John P., Tamagnan Gilles D., Barret Olivier (2018). Development and In Vivo Preclinical Imaging of Fluorine-18-Labeled Synaptic Vesicle Protein 2A (SV2A) PET Tracers. Molecular Imaging and Biology.

[CR25] Coughlin Jennifer M., Du Yong, Crawford Jeffrey L., Rubin Leah H., Azad Babak Behnam, Lesniak Wojciech G., Horti Andrew G., Schretlen David J., Sawa Akira, Pomper Martin G. (2018). Use of 18F-ASEM PET to Determine the Availability of the α7-Nicotinic Acetylcholine Receptor in Recent-Onset Psychosis. Journal of Nuclear Medicine.

[CR26] Coughlin JM, Slania S, Du Y, Rosenthal HB, Lesniak WG, Minn I (2018). ^18^F-XTRA PET for enhanced imaging of the Extrathalamic α4β2 nicotinic acetylcholine receptor. J Nucl Med.

[CR27] Crutch SJ, Lehmann M, Schott JM, Rabinovici GD, Rossor MN, Fox NC (2012). Posterior cortical atrophy. Lancet Neurol.

[CR28] Crutch SJ, Schott JM, Rabinovici GD, Murray M, Snowden JS, van der Flier WM (2017). Consensus classification of posterior cortical atrophy. Alzheimers Dement.

[CR29] Curtis C, Gamez JE, Singh U, Sadowsky CH, Villena T, Sabbagh MN (2015). Phase 3 trial of flutemetamol labeled with radioactive fluorine 18 imaging and neuritic plaque density. JAMA Neurol.

[CR30] DeKosky ST, Scheff SW (1990). Synapse loss in frontal cortex biopsies in Alzheimer's disease: correlation with cognitive severity. Ann Neurol.

[CR31] Deuther-Conrad W, Fischer S, Hiller A, Becker G, Cumming P, Xiong G (2011). Assessment of α7 nicotinic acetylcholine receptor availability in juvenile pig brain with ^18^FNS10743. Eur J Nucl Med Mol Imaging.

[CR32] Dickson DW (2018). Neuropathology of Parkinson disease. Parkinsonism Relat Disord.

[CR33] Drzezga A (2010). Amyloid-plaque imaging in early and differential diagnosis of dementia. Ann Nucl Med.

[CR34] Dubois B, Feldman HH, Jacova C, Hampel H, Molinuevo JL, Blennow K (2014). Advancing research diagnostic criteria for Alzheimer's disease: the IWG-2 criteria. Lancet Neurol.

[CR35] Dugger BN, Adler CH, Shill HA, Caviness J, Jacobson S, Driver-Dunckley E, Beach TG (2014). Concomitant pathologies among a spectrum of parkinsonian disorders. Parkinsonism Relat Disord.

[CR36] Edison P, Rowe CC, Rinne JO, Ng S, Ahmed I, Kemppainen N (2008). Amyloid load in Parkinson's disease dementia and Lewy body dementia measured with 11CPIB positron emission tomography. J Neurol Neurosurg Psychiatry.

[CR37] Ettrup A, Mikkelsen JD, Lehel S, Madsen J, Nielsen EØ, Palner M (2011). 11C-NS14492 as a novel PET radioligand for imaging cerebral alpha7 nicotinic acetylcholine receptors: in vivo evaluation and drug occupancy measurements. J Nucl Med.

[CR38] Feng G, Xiao F, Lu Y, Huang Z, Yuan J, Xiao Z (2009). Down-regulation synaptic vesicle protein 2A in the anterior temporal neocortex of patients with intractable epilepsy. J Mol Neurosci.

[CR39] Finnema SJ, Nabulsi NB, Eid T, Detyniecki K, Lin S-F, Chen M-K (2016). Imaging synaptic density in the living human brain. Sci Transl Med.

[CR40] Flynn DD, Mash DC (1986). Characterization of L-3Hnicotine binding in human cerebral cortex: comparison between Alzheimer's disease and the normal. J Neurochem.

[CR41] Friedman JH (2018). Dementia with Lewy bodies and Parkinson disease dementia: it is the same disease!. Parkinsonism Relat Disord.

[CR42] Frisoni GB, Boccardi M, Barkhof F, Blennow K, Cappa S, Chiotis K (2017). Strategic roadmap for an early diagnosis of Alzheimer's disease based on biomarkers. Lancet Neurol.

[CR43] Gao R, Zhang G, Chen X, Reid S, Zhou Y (2016). 18F-AV133 cerebral VMAT2 binding correlated with α-synuclein spliced variants in Parkinson’s disease. J Neuroimaging Psychiatry Neurol.

[CR44] Gilman S, Wenning GK, Low PA, Brooks DJ, Mathias CJ, Trojanowski JQ (2008). Second consensus statement on the diagnosis of multiple system atrophy. Neurology..

[CR45] Glantz LA, Lewis DA (2000). Decreased dendritic spine density on prefrontal cortical pyramidal neurons in schizophrenia. Arch Gen Psychiatry.

[CR46] Grimmer T, Shi K, Diehl-Schmid J, Natale B, Drzezga A, Förster S, et al. 18F-FIBT may expand PET for β-amyloid imaging in neurodegenerative diseases. Mol Psychiatry. 2018. 10.1038/s41380-018-0203-5. 10.1038/s41380-018-0203-5 PMC7515824 30120417

[CR47] Hamos JE, DeGennaro LJ, Drachman DA (1989). Synaptic loss in Alzheimer's disease and other dementias. Neurology..

[CR48] Harada R, Okamura N, Furumoto S, Yanai K (2018). Imaging protein Misfolding in the brain using β-sheet ligands. Front Neurosci.

[CR49] Harris JM, Gall C, Thompson JC, Richardson AMT, Neary D, Du Plessis D (2013). Classification and pathology of primary progressive aphasia. Neurology..

[CR50] Harris JM, Jones M (2014). Pathology in primary progressive aphasia syndromes. Curr Neurol Neurosci Rep.

[CR51] Hellwig S, Amtage F, Kreft A, Buchert R, Winz OH, Vach W (2012). ^18^FFDG-PET is superior to ^123^IIBZM-SPECT for the differential diagnosis of parkinsonism. Neurology..

[CR52] Herrera-Rivero M, Heneka MT, Papadopoulos V (2015). Translocator protein and new targets for neuroinflammation. Ckin Transl Imaging.

[CR53] Hillmer AT, Zheng M-Q, Li S, Scheunemann M, Lin S-F, Holden D (2016). PET imaging evaluation of (18) FDBT-10, a novel radioligand specific to α7 nicotinic acetylcholine receptors, in nonhuman primates. Eur J Nucl Med Mol Imaging.

[CR54] Horti AG, Kuwabara H, Holt DP, Dannals RF, Wong DF (2013). Recent PET radioligands with optimal brain kinetics for imaging nicotinic acetylcholine receptors. J Labelled Comp Radiopharm.

[CR55] Hsiao I-T, Weng Y-H, Lin W-Y, Hsieh C-J, Wey S-P, Yen T-C (2014). Comparison of 99mTc-TRODAT-1 SPECT and 18 F-AV-133 PET imaging in healthy controls and Parkinson's disease patients. Nucl Med Biol.

[CR56] Ishikawa M, Sakata M, Toyohara J, Oda K, Ishii K, Wu J (2011). Occupancy of α7 nicotinic acetylcholine receptors in the brain by Tropisetron: a positron emission tomography study using (11) CCHIBA-1001 in healthy human subjects. Clin Psychopharmacol Neurosci.

[CR57] Ito H, Shimada H, Shinotoh H, Takano H, Sasaki T, Nogami T (2014). Quantitative analysis of amyloid deposition in Alzheimer disease using PET and the radiotracer ^11^C-AZD2184. J Nucl Med.

[CR58] Jack CR, Bennett DA, Blennow K, Carrillo MC, Dunn B, Haeberlein SB (2018). NIA-AA research framework: toward a biological definition of Alzheimer's disease. Alzheimers Dement.

[CR59] Jellinger KA (2014). Neuropathology of multiple system atrophy: new thoughts about pathogenesis. Mov Disord.

[CR60] Kang HJ, Voleti B, Hajszan T, Rajkowska G, Stockmeier CA, Licznerski P (2012). Decreased expression of synapse-related genes and loss of synapses in major depressive disorder. Nat Med.

[CR61] Kendziorra K, Wolf H, Meyer PM, Barthel H, Hesse S, Becker GA (2011). Decreased cerebral α4β2* nicotinic acetylcholine receptor availability in patients with mild cognitive impairment and Alzheimer's disease assessed with positron emission tomography. Eur J Nucl Med Mol Imaging.

[CR62] Kim SD, Fung VSC (2014). An update on Huntington's disease: from the gene to the clinic. Curr Opin Neurol.

[CR63] Koole Michel, van Aalst June, Devrome Martijn, Mertens Nathalie, Serdons Kim, Lacroix Brigitte, Mercier Joel, Sciberras David, Maguire Paul, Van Laere Koen (2018). Quantifying SV2A density and drug occupancy in the human brain using [11C]UCB-J PET imaging and subcortical white matter as reference tissue. European Journal of Nuclear Medicine and Molecular Imaging.

[CR64] Krstic D, Knuesel I (2013). Deciphering the mechanism underlying late-onset Alzheimer disease. Nat Rev Neurol.

[CR65] Kuhl DE, Koeppe RA, Minoshima S, Snyder SE, Ficaro EP, Foster NL (1999). In vivo mapping of cerebral acetylcholinesterase activity in aging and Alzheimer's disease. Neurology..

[CR66] Kuhl DE, Minoshima S, Fessler JA, Frey KA, Foster NL, Ficaro EP (1996). In vivo mapping of cholinergic terminals in normal aging, Alzheimer's disease, and Parkinson's disease. Ann Neurol.

[CR67] Leenders KL, Salmon EP, Tyrrell P, Perani D, Brooks DJ, Sager H (1990). The nigrostriatal dopaminergic system assessed in vivo by positron emission tomography in healthy volunteer subjects and patients with Parkinson's disease. Arch Neurol.

[CR68] Lois Cristina, Gonzalez Ivan, Johnson Keith A., Price Julie C. (2018). PET imaging of tau protein targets: a methodology perspective. Brain Imaging and Behavior.

[CR69] Lucero C, Campbell MC, Flores H, Maiti B, Perlmutter JS, Foster ER (2015). Cognitive reserve and β-amyloid pathology in Parkinson disease. Parkinsonism Relat Disord.

[CR70] MacDonald ME, Ambrose CM, Duyao MP, Myers RH, Lin C, Srinidhi L (1993). A novel gene containing a trinucleotide repeat that is expanded and unstable on Huntington's disease chromosomes. Cell..

[CR71] Mackenzie IRA, Neumann M (2016). Molecular neuropathology of frontotemporal dementia: insights into disease mechanisms from postmortem studies. J Neurochem.

[CR72] Martin-Ruiz C, Court J, Lee M, Piggott M, Johnson M, Ballard C (2000). Nicotinic receptors in dementia of Alzheimer, Lewy body and vascular types. Acta Neurol Scand Suppl.

[CR73] Mathis CA, Lopresti BJ, Ikonomovic MD (2017). Klunk WE4. Small-molecule PET tracers for imaging Proteinopathies. Semin Nucl Med.

[CR74] McKeith I, Taylor J-P, Thomas A, Donaghy P, Kane J (2016). Revisiting DLB diagnosis: a consideration of prodromal DLB and of the diagnostic overlap with Alzheimer disease. J Geriatr Psychiatry Neurol.

[CR75] McKeith IG, Boeve BF, Dickson DW, Halliday G, Taylor J-P, Weintraub D (2017). Diagnosis and management of dementia with Lewy bodies: fourth consensus report of the DLB consortium. Neurology..

[CR76] McKeith IG, Dickson DW, Lowe J, Emre M, O'Brien JT, Feldman H (2005). Diagnosis and management of dementia with Lewy bodies: third report of the DLB consortium. Neurology..

[CR77] McKhann GM, Knopman DS, Chertkow H, Hyman BT, Jack CR, Kawas CH (2011). The diagnosis of dementia due to Alzheimer's disease: recommendations from the National Institute on Aging-Alzheimer's Association workgroups on diagnostic guidelines for Alzheimer's disease. Alzheimers Dement.

[CR78] Mesulam M-M, Weintraub S, Rogalski EJ, Wieneke C, Geula C, Bigio EH (2014). Asymmetry and heterogeneity of Alzheimer's and frontotemporal pathology in primary progressive aphasia. Brain..

[CR79] Meyer PM, Strecker K, Kendziorra K, Becker G, Hesse S, Woelpl D (2009). Reduced alpha4beta2*-nicotinic acetylcholine receptor binding and its relationship to mild cognitive and depressive symptoms in Parkinson disease. Arch Gen Psychiatry.

[CR80] Meyer PM, Tiepolt S, Barthel H, Hesse S, Sabri O (2014). Radioligand imaging of α4β2* nicotinic acetylcholine receptors in Alzheimer's disease and Parkinson's disease. Q J Nucl Med Mol Imaging.

[CR81] Meyer PT, Frings L, Rücker G, Hellwig S (2017). ^18^F-FDG PET in parkinsonism: differential diagnosis and evaluation of cognitive impairment. J Nucl Med.

[CR82] Minoshima S, Frey KA, Cross DJ, Kuhl DE (2004). Neurochemical imaging of dementias. Semin Nucl Med.

[CR83] Mueller A, Kroth H, Berndt M, Capotosti F, Molette J, Schieferstein H (2017). Characterization of the novel PET Tracer PI-2620 for the assessment of Tau pathology in Alzheimer’s disease and other tauopathies. J Nucl Med.

[CR84] O'Brien JT, Colloby SJ, Pakrasi S, Perry EK, Pimlott SL, Wyper DJ (2007). Alpha4beta2 nicotinic receptor status in Alzheimer's disease using ^123^I-5IA-85380 single-photon-emission computed tomography. J Neurol Neurosurg Psychiatry.

[CR85] Ory D, Celen S, Verbruggen A, Bormans G (2014). PET radioligands for in vivo visualization of neuroinflammation. Curr Pharm Des.

[CR86] Perry DC, Brown JA, Possin KL, Datta S, Trujillo A, Radke A (2017). Clinicopathological correlations in behavioural variant frontotemporal dementia. Brain..

[CR87] Perry EK, Morris CM, Court JA, Cheng A, Fairbairn AF, McKeith IG (1995). Alteration in nicotine binding sites in Parkinson's disease, Lewy body dementia and Alzheimer's disease: possible index of early neuropathology. Neuroscience..

[CR88] Postuma RB, Berg D, Stern M, Poewe W, Olanow CW, Oertel W (2015). MDS clinical diagnostic criteria for Parkinson's disease. Mov Disord.

[CR89] Pressman PS, Miller BL (2014). Diagnosis and management of behavioral variant frontotemporal dementia. Biol Psychiatry.

[CR90] Renner JA, Burns JM, Hou CE, McKeel DW, Storandt M, Morris JC (2004). Progressive posterior cortical dysfunction: a clinicopathologic series. Neurology..

[CR91] Respondek G, Roeber S, Kretzschmar H, Troakes C, Al-Sarraj S, Gelpi E (2013). Accuracy of the National Institute for neurological disorders and stroke/Society for Progressive Supranuclear Palsy and neuroprotection and natural history in Parkinson plus syndromes criteria for the diagnosis of progressive supranuclear palsy. Mov Disord.

[CR92] Rogalski E, Cobia D, Martersteck A, Rademaker A, Wieneke C, Weintraub S, Mesulam M-M (2014). Asymmetry of cortical decline in subtypes of primary progressive aphasia. Neurology..

[CR93] Rowe CC, Pejoska S, Mulligan RS, Jones G, Chan JG, Svensson S (2013). Head-to-head comparison of 11C-PiB and 18F-AZD4694 (NAV4694) for β-amyloid imaging in aging and dementia. J Nucl Med.

[CR94] Roy R, Niccolini F, Pagano G, Politis M (2016). Cholinergic imaging in dementia spectrum disorders. Eur J Nucl Med Mol Imaging.

[CR95] Sabri O, Becker GA, Meyer PM, Hesse S, Wilke S, Graef S (2015). First-in-human PET quantification study of cerebral α4β2* nicotinic acetylcholine receptors using the novel specific radioligand (−)-[^18^F]Flubatine. Neuroimage..

[CR96] Sabri O, Kendziorra K, Wolf H, Gertz HJ, Brust P (2008). Acetylcholine receptors in dementia and mild cognitive impairment. Eur J Nucl Med Mol Imaging.

[CR97] Sabri O, Meyer PM, Gräf S, Hesse S, Wilke S, Becker G-A (2018). Cognitive correlates of α4β2 nicotinic acetylcholine receptors in mild Alzheimer's dementia. Brain..

[CR98] Sabri O, Sabbagh MN, Seibyl J, Barthel H, Akatsu H, Ouchi Y (2015). Florbetaben PET imaging to detect amyloid beta plaques in Alzheimer's disease: phase 3 study. Alzheimers Dement.

[CR99] Seibyl JP (2008). Single-photon emission computed tomography and positron emission tomography evaluations of patients with central motor disorders. Semin Nucl Med.

[CR100] Sihver W, Gillberg PG, Svensson AL, Nordberg A (1999). Autoradiographic comparison of ^3^H(−)nicotine, ^3^Hcytisine and ^3^Hepibatidine binding in relation to vesicular acetylcholine transport sites in the temporal cortex in Alzheimer's disease. Neuroscience..

[CR101] Skogseth R, Hortobágyi T, Soennesyn H, Chwiszczuk L, Ffytche D, Rongve A (2017). Accuracy of clinical diagnosis of dementia with Lewy bodies versus neuropathology. J Alzheimers Dis.

[CR102] Sultzer DL, Melrose RJ, Riskin-Jones H, Narvaez TA, Veliz J, Ando TK (2017). Cholinergic receptor binding in Alzheimer disease and healthy aging: assessment in vivo with positron emission tomography imaging. Am J Geriatr Psychiatry.

[CR103] Svetoni F, Frisone P, Paronetto MP (2016). Role of FET proteins in neurodegenerative disorders. RNA Biol.

[CR104] Tang CC, Feigin A, Ma Y, Habeck C, Paulsen JS, Leenders KL (2013). Metabolic network as a progression biomarker of premanifest Huntington's disease. J Clin Invest.

[CR105] Tang-Wai DF, Graff-Radford NR, Boeve BF, Dickson DW, Parisi JE, Crook R (2004). Clinical, genetic, and neuropathologic characteristics of posterior cortical atrophy. Neurology..

[CR106] Toyohara J, Ishiwata K, Sakata M, Wu J, Nishiyama S, Tsukada H, Hashimoto K (2010). In vivo evaluation of alpha7 nicotinic acetylcholine receptor agonists ^11^CA-582941 and ^11^CA-844606 in mice and conscious monkeys. PLoS One.

[CR107] van Vliet EA, Aronica E, Redeker S, Boer K, Gorter JA (2009). Decreased expression of synaptic vesicle protein 2A, the binding site for levetiracetam, during epileptogenesis and chronic epilepsy. Epilepsia..

[CR108] Verdurand M, Levigoureux E, Zeinyeh W, Berthier L, Mendjel-Herda M, Cadarossanesaib F (2018). In silico, in vitro, and in vivo evaluation of new candidates for α-Synuclein PET imaging. Mol Pharm.

[CR109] Villemagne VL (2018). Selective Tau Imaging: Der Stand der Dinge. J Nucl Med.

[CR110] Villemagne VL, Fodero-Tavoletti MT, Masters CL, Rowe CC (2015). Tau imaging: early progress and future directions. Lancet Neurol.

[CR111] Vuono R, Winder-Rhodes S, de Silva R, Cisbani G, Drouin-Ouellet J, Spillantini MG (2015). The role of tau in the pathological process and clinical expression of Huntington's disease. Brain..

[CR112] Wang H-Y, Li W, Benedetti NJ, Lee DHS (2003). Alpha 7 nicotinic acetylcholine receptors mediate beta-amyloid peptide-induced tau protein phosphorylation. J Biol Chem.

[CR113] Wong Dean F., Comley Robert A., Kuwabara Hiroto, Rosenberg Paul B., Resnick Susan M., Ostrowitzki Susanne, Vozzi Cristina, Boess Frank, Oh Esther, Lyketsos Constantine G., Honer Michael, Gobbi Luca, Klein Gregory, George Noble, Gapasin Lorena, Kitzmiller Kelly, Roberts Josh, Sevigny Jeff, Nandi Ayon, Brasic James, Mishra Chakradhar, Thambisetty Madhav, Moghekar Abhay, Mathur Anil, Albert Marilyn, Dannals Robert F., Borroni Edilio (2018). Characterization of 3 Novel Tau Radiopharmaceuticals, 11C-RO-963, 11C-RO-643, and 18F-RO-948, in Healthy Controls and in Alzheimer Subjects. Journal of Nuclear Medicine.

[CR114] Wong DF, Kuwabara H, Kim J, Brasic JR, Chamroonrat W, Gao Y (2013). PET imaging of high-affinity α4β2 nicotinic acetylcholine receptors in humans with ^18^F-AZAN, a radioligand with optimal brain kinetics. J Nucl Med.

[CR115] Wong DF, Kuwabara H, Pomper M, Holt DP, Brasic JR, George N (2014). Human brain imaging of α7 nAChR with [^18^F]ASEM: a new PET radiotracer for neuropsychiatry and determination of drug occupancy. Mol Imaging Biol.

